# Development and Validation of a Spectrofluorimetric Method for the Estimation of Rivastigmine in Formulations

**DOI:** 10.4103/0250-474X.58179

**Published:** 2009

**Authors:** R. Kapil, S. Dhawan, Bhupinder Singh

**Affiliations:** University Institute of Pharmaceutical Sciences (UGC Centre of Advanced Studies) Panjab University, Chandigarh-160 014, India

**Keywords:** Detection limit, fluorescence spectrophotometry, fluorimetry, linearity, quantitation limit, validation

## Abstract

A rapid, sensitive, simple, and cost-effective spectrofluorimetric method was developed for the estimation of rivastigmine in bulk and pharmaceutical formulations. The relative fluorescence intensity of rivastigmine was measured in triple distilled water at an excitation wavelength of 220 nm and an emission wavelength of 289 nm. Linearity range was found to be 100 to 4000 ng/ml. The method was validated for various parameters as per the ICH guidelines and USP requirements. The detection and quantitation limits were found to be 20.5 and 62.1 ng/ml, respectively. The results demonstrate that the procedure is accurate, precise, and reproducible, while being simple and rapid too. The results were found to be in good agreement with the label claims.

Rivastigmine has been available as the drug of choice for the symptomatic treatment of moderate to severe Alzheimer's disease. More recently, it has been indicated in mild to moderate dementia associated with Parkinson disease too. The usage of rivastigmine has been approved in capsule and liquid form in several countries like US and UK since 1997. Rivastigmine is a dual inhibitor of acetylcholinesterase and butyrylcholinesterase. Its efficacy is dose-related, with daily oral doses ranging between 6 mg and 12 mg[1]. Owing to its numerous clinical advantages, there has been a spurt in the number of publications on rivastigmine, esp. on its formulation aspects[2–4].

Increasing popularity of rivastigmine, therefore, necessitates the development of a simple analytical method for its estimation in bulk and formulations, and during dissolution runs. UV/Vis spectrophotometry, in this context, is of limited utility because of its non-specific λ_max_ i.e., 221 nm, a region of high spectrophotometric interference. High performance liquid chromatography (HPLC) methods utilizing UV and fluorescence detectors are reported in literature[5,6] for estimation of rivastigmine in dissolution release media and in biological fluids like plasma and serum. The chromatographic techniques, however, demand a lot of time, cost and expertise in their operation.

The objective of the present study, therefore, was to develop a simple, sensitive, rapid, precise, accurate, effective and cost-effective analytical method for estimation of rivastigmine in pharmaceutical formulations and during *in vitro* dissolution studies of its formulations. Further, the study would embark upon the validation of the developed methodology as per the ICH guidelines[7] and USP requirements[8].

Rivastigmine hydrogen tartarate was obtained *ex gratis* from M/s Sun Pharma Ltd, Vadodara, India and M/s Cipla Pharma Ltd, Mumbai, India. Marketed brand (Rivamer® 1.5 mg, Batch no. GK71822, Sun Pharma Ltd, Kartholi, J&K, India) was employed as the reference. All other chemicals and reagents were of analytical grade and were employed as such. All the fluorescence measurements were conducted on a spectrofluorimeter (Hitachi F 2500, Japan) equipped with a Xenon arc lamp, preloaded with a data interpreting software (FL Solutions ver. 2.0).

Various dissolution media *viz*. distilled water, 0.1N HCl, phosphate buffer (pH 6.8) and normal saline, alone and in combination with different organic solvents, in various proportions, were employed based on the sensitivity, ease of sample preparation, drug solubility, cost and applicability of the method employed. The relative fluorescence intensity (RFI) of rivastigmine was measured at an excitation wavelength (i.e., activating wavelength, λ_exc_) of 220 nm and an emission wavelength (i.e., fluorescence wavelength, λ_em_) of 289 nm. Fig. 1 depicts a scan of the emission fluorescence of the drug obtained at the λ_exc_ of 220 nm. The slit width for excitation and emission was kept as 10 nm. The photo-multiplier tube voltage was set at 700 V. Primary stock solution of 1000 μg/ml of rivastigmine hydrogen tartarate was prepared in triple distilled water (TDW). Secondary stock solution of 10 μg/ml of drug was prepared in TDW using aliquots of primary stock solution. For preparation of different drug concentrations, aliquots of secondary stock solution were transferred into a series of 10 ml standard flasks and volume was made up with TDW. A total of 12 different concentrations (100, 150, 200, 250, 300, 400, 500, 1000, 2000, 2500, 3000 and 4000 ng/ml) of rivastigmine were prepared for constructing a standard calibration curve and their RFI was recorded against blank.

**Fig. 1:**
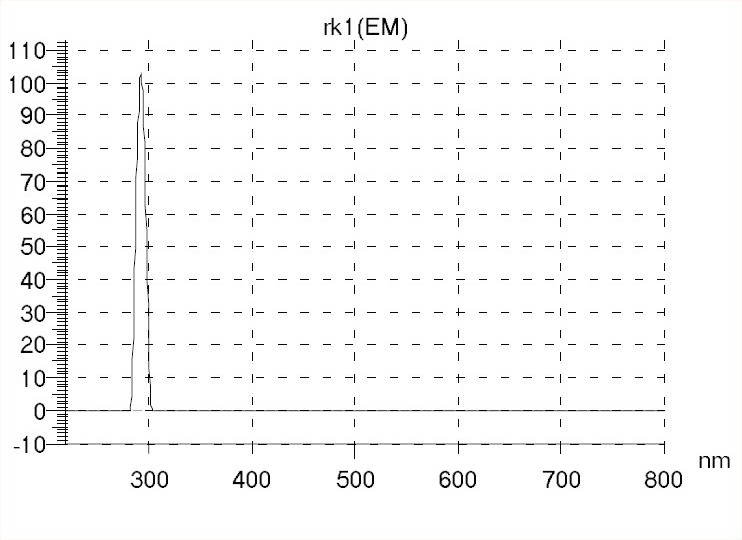
Emission scan of rivastigmine at the excitation wavelength of 220 nm

To establish linearity of the proposed method, six separate series of solutions of the drug in the selected medium were prepared from the stock solution and analyzed. Least square linear regression analysis was conducted on the obtained spectrofluorimetric data using MS-Excel 2007 spreadsheet software. Different concentrations and their relative fluorescence intensities are shown in Table 1. At all the drug concentration levels studied, the values of standard deviation (SD < 7.8%) and the relative standard deviation (RSD < 3.9%) were found to be quite low, indicating high repeatability. The values of predicted concentrations were nearly matching with that of the nominal observed concentrations.

**TABLE 1:** CALIBRATION DATA OF RIVASTIGMINE

Drug concentration(ng/ml)	Mean relative fluorescence intensity (±SD)	% RSD	Predicted drug concentration (ng/ml)
100	47.3±0.66	1.405	100.8
150	65.7±0.62	0.946	146.7
200	85.3±0.69	0.812	195.4
250	105.9±3.02	2.891	246.7
300	122.1±4.89	3.995	287.0
400	172.8±5.09	2.946	413.2
500	202.6±4.52	2.233	487.4
1000	400.1±7.00	1.749	979.1
2000	812.8±5.79	0.713	2006.4
2500	1021.1±7.78	0.761	2524.7
3000	1217.9±7.07	0.580	3015.2
4000	1604.2±7.07	0.440	3976.1

Linearity of the method was confirmed by plotting the ratio of response: concentration (i.e., sensitivity) *vs*. log of concentration[9]. The linearity in the selected medium (TDW) was found to range between 100 and 4000 ng/ml. The graphical plot between sensitivity (response/amount) and log concentration also exhibited linearity in the said range, as depicted in fig. 2. Rivastigmine solutions (200 ng/ml) were prepared in the selected medium with and without common excipients (lactose, starch, methylcellulose, hydroxypropylmethylcellulose). All the studied solutions were scanned for their emission spectra at a fixed λ_exc_ of 220 nm and investigated for the change in emission spectrum, if any. The emission spectrum of rivastigmine was not found to alter in the presence of these common excipients in the selected medium. No statistically significant difference in the relative fluorescence intensity was observed between identical concentrations of pure drug sample and that of sample with excipients (P>0.05). Hence, insignificant interference of excipients during the estimation of drug could be inferred. The proposed method, therefore, was found to be quite specific and selective for the drug which could be potentially employed for its estimation in pharmaceutical formulations. The spectrophotometric method at a λ_max_ of 221 nm, on the contrary, was found to be quite non-specific for the drug, as various excipients and solvents also absorb significantly in the said region.

**Fig. 2:**
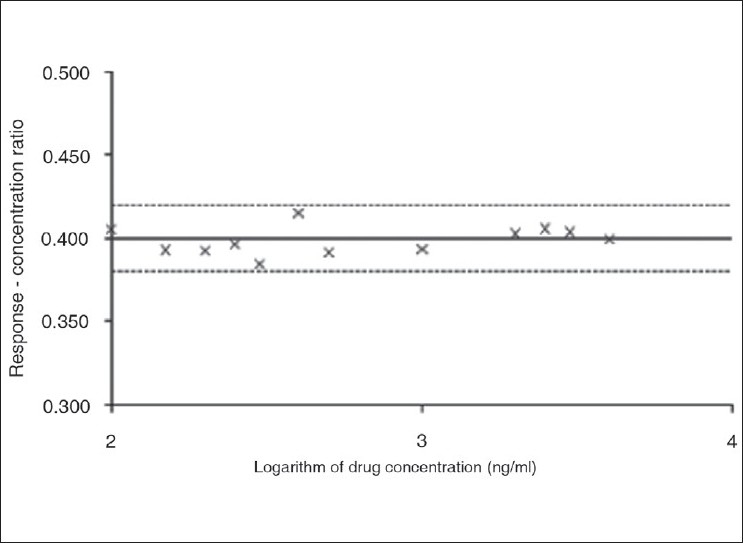
Validation of linearity of the analytical method of rivastigmine

To determine the accuracy of the proposed method, different quality control solutions, i.e., low (LQC: 150), medium (MQC: 500), and high (HQC: 2500 ng/ml) were prepared independently from stock solution and analyzed (n=6). Accuracy was assessed as the percentage relative error and mean percentage recovery[10]. The magnitudes of prediction error (i.e., bias) values ranged between −0.18 and 0.16% for the three concentration levels studied (Table 2), unequivocally vouching high accuracy of the methodology employed. Further, as indicated in the table, the high mean % recovery values (nearly 100%) and the corresponding low standard deviation values (≤0.98%) observed during the studies also corroborated high accuracy of the method.

**TABLE 2:** ACCURACY DATA FOR THE DEVELOPED ANALYTICAL METHOD

Level	Predicted concentration (ng/ml)			Mean % recovery (± SD)	Bias (%)
			
	Range	Mean (± SD)	% RSD
LQC	148.0-150.9	149.7±1.47	0.983	99.82±0.98	− 0.18
MQC	494.2-503.1	500.3±4.40	0.880	100.06±0.88	0.06
HQC	2495.3-2512.2	2504.3±8.29	0.331	100.16±0.33	0.16

Repeatability was determined using different levels of drug concentrations (as above during determination of accuracy), prepared from independent stock solution and analyzed (n=6). Inter-day variation and intra-day variation, and analyst variation were studied to determine intermediate precision of the proposed method. Different levels of drug concentrations (in triplicate) were prepared at two different times in a day and studied for intra-day variation. The identical protocol was followed on three different days to study inter-day variation (n=18). Different analysts prepared different solutions on different days. The RSD of the predicted concentrations from the regression equation was taken as the value of precision. In the repeatability study, the RSD values ranged between 0.331 and 0.983% (Table 2). At all the three studied concentration levels, precision showed satisfactory levels. Intermediate precision expresses within-laboratory variation on different days and by different analysts. Results of intermediate precision study and RSD values for each set at all the three levels are enlisted in Table 3. In all the cases, low magnitude of RSD (< 1.00%) observed in the studies construe excellent repeatability and intermediate precision of the method.

**TABLE 3:** DATA DEPICTING INTERMEDIATE PRECISION STUDY

Level	Intra-day repeatability, % RSD (n = 3)				Inter-day repeatability, % RSD (n = 18)
		
	Analyst	Day 1	Day 2	Day 3	
LQC	1	0.833	0.784	0.973	0.971
	2	0.925	0.902	0.663	
MQC	1	0.529	0.564	0.871	0.762
	2	0.806	0.781	0.691	
HQC	1	0.410	0.448	0.487	0.396
	2	0.323	0.215	0.428	

The values of detection limit (DL) and quantitation limit (QL) of rivastigmine by the proposed method was calculated using the standard calibration curve as 3.3 σ/*S* and 10 σ/*S*, respectively, where, *S* is the slope of the calibration curve and σ is the standard deviation of the response. The values of DL and QL for rivastigmine were found to be 20.5 and 62.1 ng/ml, respectively. Evidently, this indicated excellent sensitivity of the method even at sub-microgram levels. In contrast, the values of DL and QL for spectrophotometric method were found to be quite high, i.e., 0.93 and 2.82 μg/ml, respectively.

Robustness of the proposed method was determined by changing pH of the media by ± 0.2 units and analyzing stability of drug in the selected medium at room temperature for 10 h. Three different concentrations (LQC, MQC and HQC) were prepared in different pH media and mean percentage recovery was determined[10]. Robustness was found to be quite high, as the variation of pH of the selected media by ± 0.2 did not have any significant effect on RFI values. Mean percentage of recovery (±SD) was found to be 100.37% (±1.29). Drug solution in the selected medium exhibited no spectrofluorimetric change(s) for 10 h, when kept at room temperature.

The proposed method was also evaluated by estimation of rivastigmine in the pharmaceutical formulations. Extraction of drug from the formulation, or otherwise, was considered unnecessary; hence was not employed. Twenty capsules (Rivamer® 1.5 mg) were weighed and emptied on a butter paper. Amount of the powder equivalent to 1.5 mg of rivastigmine was taken, dissolved in the selected medium and filtered. The solution was diluted suitably to prepare a concentration of 1.5 μg/ml of drug. This primary stock solution was filtered through Whatman® filter paper and the filtrate was further diluted to prepare a solution of 150 ng/ml of rivastigmine. The RFI value of the solution, thus prepared, was observed to estimate the total rivastigmine content in the formulation. The assay values of three samples of Rivamer® capsules ranged between 98.23 and 101.76%. Assay values (1.47–1.52 mg) of formulations were found to be quite close to the label claim of 1.5 mg. This corroborated that the interference of excipient matrix is insignificant in the estimation of rivastigmine by the proposed method. The whole process of assay involved the expenditure of minimal time (sparing a few min) and money. In contrast, HPLC procedures for the routine drug analysis require a great deal of developmental effort and expenditure in terms of solvents, columns, guard columns, productive and non-productive time etc. Hence, the spectrofluorimetric method vouches its undisputed efficacy, both in terms of time and cost.

In a nutshell, the proposed method was found to be sensitive, simple, rapid, accurate, precise and inexpensive for routine analysis of rivastigmine in bulk, pharmaceutical formulations and during dissolution studies of oral formulations. The sample recoveries in all the investigated formulations were in good agreement with their respective label claims, indicating non-interference of excipients during the spectrofluorimetric estimation of drug.
